# The sequence and analysis of a Chinese pig genome

**DOI:** 10.1186/2047-217X-1-16

**Published:** 2012-11-15

**Authors:** Xiaodong Fang, Yulian Mou, Zhiyong Huang, Yong Li, Lijuan Han, Yanfeng Zhang, Yue Feng, Yuanxin Chen, Xuanting Jiang, Wei Zhao, Xiaoqing Sun, Zhiqiang Xiong, Lan Yang, Huan Liu, Dingding Fan, Likai Mao, Lijie Ren, Chuxin Liu, Juan Wang, Kui Li, Guangbiao Wang, Shulin Yang, Liangxue Lai, Guojie Zhang, Yingrui Li, Jun Wang, Lars Bolund, Huanming Yang, Jian Wang, Shutang Feng, Songgang Li, Yutao Du

**Affiliations:** 1 BGI-Shenzhen, Bei Shan Road, Yantian, Shenzhen, 518083, China; 2 Institute of Animal Science (IAS), Chinese Academy of Agriculture Science (CAAS), Beijing, 10094, China; 3 Shenzhen Engineering Laboratory for Genomics-Assisted Animal Breeding, BGI-Shenzhen, Bei Shan Road, Yantian, Shenzhen, 518083, China; 4 BGI Ark Biotechnology (BAB), Bei Shan Road, Yantian, Shenzhen, 518083, China; 5 State Key Laboratory of Genetic Resources and Evolution, Kunming Institute of Zoology, Chinese Academy of Sciences, Kunming, China; 6 Department of Neurology, Shenzhen Second People’s Hospital (First Affiliated Hospital of Shenzhen University), Shenzhen, 518035, China; 7 Guangzhou Institute of Biomedicine and Health, Chinese Academy of Sciences, Guangdong, China; 8 Novo Nordisk Foundation Center for Basic Metabolic Research, University of Copenhagen, Copenhagen, Denmark; 9 Department of Biology, University of Copenhagen, Copenhagen, Denmark; 10 Department of Biomedicine, Aarhus University, Aarhus C, Denmark

**Keywords:** Wuzhishan pig, Genome, Homozygosis, Transposable element, Endogenous retrovirus, Animal model

## Abstract

**Background:**

The pig is an economically important food source, amounting to approximately 40% of all meat consumed worldwide. Pigs also serve as an important model organism because of their similarity to humans at the anatomical, physiological and genetic level, making them very useful for studying a variety of human diseases. A pig strain of particular interest is the miniature pig, specifically the Wuzhishan pig (WZSP), as it has been extensively inbred. Its high level of homozygosity offers increased ease for selective breeding for specific traits and a more straightforward understanding of the genetic changes that underlie its biological characteristics. WZSP also serves as a promising means for applications in surgery, tissue engineering, and xenotransplantation. Here, we report the sequencing and analysis of an inbreeding WZSP genome.

**Results:**

Our results reveal some unique genomic features, including a relatively high level of homozygosity in the diploid genome, an unusual distribution of heterozygosity, an over-representation of tRNA-derived transposable elements, a small amount of porcine endogenous retrovirus, and a lack of type C retroviruses. In addition, we carried out systematic research on gene evolution, together with a detailed investigation of the counterparts of human drug target genes.

**Conclusion:**

Our results provide the opportunity to more clearly define the genomic character of pig, which could enhance our ability to create more useful pig models.

## Background

Domestic pigs belong to the mammalian clade Artiodactyla, a group of even-toed, hoofed animals whose extant representatives include ruminants such as cattle and sheep. In contrast to ruminants, pigs are omnivores and can easily adapt to changes in diet, and possess a digestive system that is simple, anatomically and physiologically distinct from ruminant stomachs. As one of the oldest forms of livestock, pigs were domesticated as early as 8,000-10,000 BC from Eurasian wild boars [1]. Pigs are commonly raised for meat, which is the most important animal protein food source, and feed a majority of the global population. Other pig derivatives include industrial materials such as pharmaceutical-grade heparin, which is mostly derived from mucosal tissues of the pig small intestine.

**Table 1:** Global statistics of the pig genome

			
**(A) Sequencing**			
	**Insert size**	**Total data (Gb)**	**Sequence coverage (X)**
Pair end library	170 ~ 800 bp	149	55
	2 ~ 20 kb	62	23
	Total	211	78
**(B) Assembly**			
	**N50 (kb)**	**Longest (kb)**	**Size (Gb)**
Contig	23.5	230	2.57
Scaffold	5,432	21,409	2.64
**(C) Annotation**			
	**Number**	**Total length (Mb)**	**Percentage of genome**
Repeats	4,172,488	1,008.6	38.2
Genes	20,326	597.3	22.6
CDS	181,843	31.2	1.2

CDS, Coding sequences.

Unlike other domesticated animals such as cattle and sheep, pigs were mainly raised in agricultural societies and settled farming communities, rather than by nomadic people. After a long period of breeding outdoors in yards or fields, pigs evolved eating patterns resembling those of human beings. The similarity dietary structure, as well as the close resemblance of pigs' digestive organs to those in humans (i.e., the stomach, pancreas and small intestine), enable pigs to develop digestion, nutrient absorption, metabolism and intestinal microflora in common with human beings. Pigs also share a number of diseases with humans, such as obesity, atherosclerosis, cardiovascular disease, gastroenteropathy and immunological diseases [2-4]. It is therefore possible to consider the pig as a unique pharmacology and toxicology model for the investigation of human health. Until now, pigs have been employed in studies involving 38 kinds of human disease, including cardiovascular and metabolic diseases [2]. In addition, pigs and humans share similarities in the size of organs as well as various other aspects of anatomy and physiology, making pigs the most promising candidate for development of new surgical procedures, tissue engineering techniques and xenotransplantation [5,6].

Of all members of the pig family, the miniature pig contains significant breeding and handling advantages, and has been proven to be particularly valuable in biomedical research. There are 72 native breeds of pig in China, accounting for about one third of worldwide breeds, including four indigenous miniature pigs, the Wuzhishan, Xiang, Diannan small-ear and Tibetan breeds [7]. This exuberant resource of pig breeds provides a large variety of genotypes and phenotypes, facilitating the use of pigs as models of different human diseases. Among these species, WZSP, characterized by its small adult size with a mature body weight of only approximately 30 kg, is one of the rare and endangered breeds previously distributed in the mountain area of Hainan province, China. Since 1987, the Institute of Animal Science of the Chinese Academy of Agriculture Science (CAAS) has developed a highly inbred strain based on the inbreeding of one male and one female WZSP by full-sib mating [8]. This inbred strain of miniature pig, with a relatively high level of homozygosis and genetic stability, provides us with genetically identical test animals to achieve good reproducibility in laboratory experiments. However, the detailed genomic structure of this strain is still unknown. Since a clear genetic background is of crucial importance in developing an effective animal model, although the genome sequence of Duroc swine generated by the International Swine Genome Sequencing Consortium (SGSC) has been publicly available for years [9], it is still short of in-depth analysis and understanding of the genome.

Here we report the sequencing and analysis of an inbreeding WZSP genome, which reveals unique genomic features, including the over representation of tRNA-derived transposable elements consisting of approximately 2.2 million copies accounting for 12.4% of the genome, as well as a relatively high degree of homozygosis of the diploid genome and its unusual distribution of heterozygosis. In addition, we investigated the counterparts of human drug target genes and genes associated with disease in the pig. Our analysis reveals that the pig resembles human closely, but attention should be drawn to the differences between human and pig when using pigs as an animal model. The reported genome, together with our detailed analysis, sheds light on our understanding of the pig genome and its evolution, increasing our understanding of human health, and enhancing possibilities for the creation of useful pig models.

## Data description

Genomic DNA was isolated from peripheral blood of a male WZSP and then used to construct Solexa libraries with various insert sizes ranging from 170 bp to 20 Kbp (including 170 bp, 350 bp, 500 bp, 800 bp, 2 Kbp, 5 Kbp, 10 Kbp and 20 Kbp). The length of reads generated from short insert size libraries (170–800 bp) and large insert size libraries (>2 Kbp) were 100 bp and 50 bp respectively. A total of 340 Gbp or 126-fold coverage of raw paired-end data were generated from these libraries. After removing the duplicated and low quality reads, 210 Gbp or 78-fold coverage of data was retained for assembly (Table 1, Supplementary Text 1.1, Table S1 in Additional file 1). Kmer-based analysis estimated the size of genome at approximately 2.5 Gbp with a very low signature of heterozygosity of the diploid genome. The high quality reads were collected and assembled using SOAPdenovo [10]. Reads from short-insert size libraries were used to build contigs and all libraries were then used for scaffolding, from smallest to largest insert-size libraries, in a stepwise process. Gaps in the scaffolds were filled by unambiguous local assembly of unmapped reads with one end uniquely mapped to the flanking regions of the gaps (Supplementary text 1.3 in Additional file 1). This process resulted in an assembly of 2.6 Gbp, and the N50 size of contigs and scaffolds were 23.5 Kbp and 5.4 Mbp, respectively Table 1. To assess the completeness of the assembly, reads from short-insert size libraries were re-mapped onto the assembly. Approximately 98% of the reads were mappable, suggesting our assembly is complete and that most of the data were represented. Supporting data, genome assemblies and annotation files are available from the GigaScience database [11].

## Analysis

### Heterozygosis of inbred diploid

Twenty generations of inbreeding should result in a high-level homozygosis of the diploid genome, but a previous study of this pedigree identified a certain genomic region where a high rate of polymorphism was maintained [12]. With the genome sequence in hand, we were able to investigate genomic regions with unusually high rates of homozygosis or heterozygosis. Polymorphism in the diploid genome, including single nucleotide polymorphism (SNP) and short insertions and deletions (short InDels) were identified by investigating the short read alignment, using the assembly as a reference. We detected 2.8 M SNPs and 392 K short InDels, resulting in a heterozygous SNP rate of 0.118% and short InDel rate of 0.017% (a combined rate of 0.135%), slightly higher than in human genome and lower than a highly inbred Iberian pig [13]. Although it is well known that the genomic diversity of pigs in China is higher than in other populations [14], considering the inbreeding, the diversity within the WZSP is unexpectedly high. We further analyzed 17 inbred mice [15] and found correlation coefficients (*r*) between observed heterozygosis and expected heterozygosis based on estimated inbreeding coefficients that are close to zero (*r* < 0.05). Although a simple examination, it suggests that estimating heterozygosis based on pedigree-derived inbreeding coefficients might not be a very good indicator of genomic heterozygosis, since genomic heterozygosis could be subject to various factors such as mutagenesis, recombination, selection and gene flow among other demographic factors.

**Figure 1:**
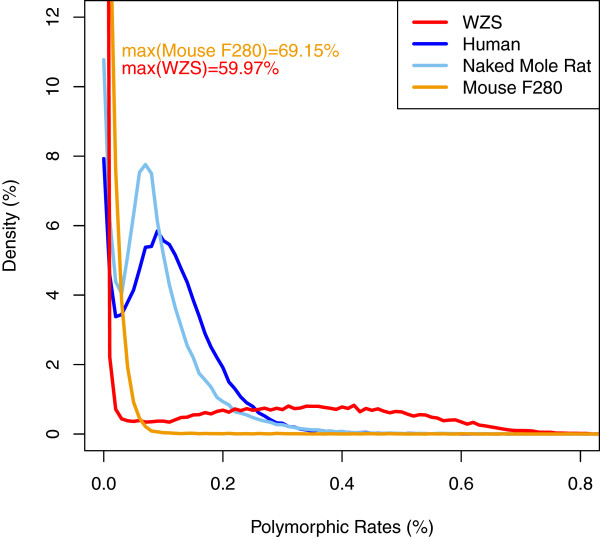
**Heterozygosis distribution of WZSP, naked mole rat, inbred mouse at F280 and human using a 50 Kbp non-overlapping sliding window.** WZSP and inbred mouse show a large amount of a low heterozygosis region (59.97% and 69.15% of the windows respectively for heterozygosis ratio < 0.01%).

The distribution of heterozygosis along the assembly was studied by a 50 Kbp non-overlapping sliding window. For comparison, the heterozygosis of human, naked-mole rat (NMR) genomes [16] and the inbred mouse at F280 [15] were also investigated. Human beings are outbred, while NMRs are proposed to be naturally inbred due to their eusocial behavior; a colony is produced by a queen mating with a few breeding males. The distribution of heterozygosis in WZSP is quite different compared to human and NMR genomes, but more like the inbred mouse genome (Figure 1). The heterozygosis between two haploids of human, NMR, WZSP and inbred mouse were 0.10%, 0.07% [16], 0.13%, and 0.01% respectively. Although it is the highest in WZSP, 60% of the WZSP genome showed extremely low heterozygosis (< 0.01%), compared with only 11% in NMR and 8% in human, suggesting that inbreeding has purged a large proportion of heterozygosity. Most regions of the human and NMR genomes share a similar rate of heterozygosis, a sharp contrast to the WZSP genome where it varied extensively, indicating that the effect of purification by inbreeding varies between different genomic loci. Highly heterozygous regions imply resistance to purification during the inbreeding process, which may be due to recombination, genetic drift and mutation, or other unknown mechanisms. One possibility is that homozygosis in these regions may lead to failed fertility, abnormal development or death, and thus excluded from our observation. Investigation of these genomic regions was performed to shed light on which genes remained heterozygous during inbreeding. We first focused on the top 5% of divergent regions with a heterozygous rate ranging from 0.55 - 1%, and found that 795 genes were shown to be located within these regions, and genes annotated with binding function were significantly enriched (p-value < 0.05). We also calculated the heterozygous rate of each coding gene, and a functional category enrichment analysis was performed focusing on the 1,000 most heterozygous genes with a heterozygosis of at least 0.48%. Olfactory receptors, G-protein coupled receptors, and other genes involved in signaling pathways were significantly over represented in this gene set (Table S3 in Additional file 1). The high divergence of genes in signaling pathways may be needed to maintain the elaborate and sophisticated regulating system, and thus cannot be purged during inbreeding. Since both recombination and selection have influence on heterozygosis, we studied the correlation between recombination and heterozygosis in these high-heterozygosis genes and found no observable correlation (correlation coefficient < 0.05). A further population study of this family will enable us to distinguish whether such heterozygous genes resulted from selection or recombination precisely.

Among the 2.8 M total SNPs identified, 20 K, 616 K and 2.16 M were located in the exon, intron and intergenic regions, resulting in SNP rates of 0.07%, 0.11% and 0.12% respectively (Table S4 in Additional file 1). The SNP rate in coding regions was much lower compared to non-coding regions, consistent with selection constraint. The ratio of transition/transversion was 2.54 and the ratio of synonymous to nonsynonymous SNPs was 0.44, comparable to human and other mammalian genomes. For the 39 K short InDels, only 348 (or 0.09%) were located in coding sequences (CDS) affecting a total of 311 genes. It is reasonable that less InDels are present in CDS since they may lead to loss-of-function due to frame shift in the triplet-based codon. However, we found 202 frame-shift mutations caused by InDels, most of which were associated with olfactory receptors, suggesting fast evolution and a high rate of gene birth and death within this category.

### Transposable elements in the pig genome

Transposable elements (TEs) are known to be well represented in vertebrate genomes and play an important role in shaping genome structure. Their high activity in transposition and recombination usually leads to structural variations, and is associated with gene birth and death. Therefore, it is vital to understand the characteristics of repeat elements in a newly sequenced genome. Based on the mechanism of transposition, TEs can be classified into either DNA transposons, which result from duplication, or retrotransposons, which are generated through retrotranscription via an RNA intermediate. Retrotransposons can be further classified into long terminal repeats (LTRs), long interspersed elements (LINEs) and short interspersed elements (SINEs).

Repetitive elements in pigs have been extensively documented through limited data or via experimental methodology [17,18]. By searching against the RepBase using Repeatmasker [19], 38.2% of the assembly was identified as TEs, more than horse (~ 32%) but less than cattle (~ 42%). Retrotransposons are the most prevalent TEs and constitute 36% of the WZSP genome, while only 2.2% of the genome was identified as DNA transposons. Among the retrotransposons, 18% of the genome consisted of LINEs, making it the most abundant category in the pig genome, but the proportion is still less than that in cattle and horse genomes (Table S5 in Additional file 1). Similar to other mammalian genomes, LINE/L1 is the most abundant class in the pig genome, accounting for 16.8% of the total genome size, comparable to 17% in horse and 18% in human, but slightly more than the 12% present in cattle. Notably, SINEs occupy 13.6% of the pig genome, making it the most SINE-rich species comparing to cattle (9.7%), horse (2.5%) and human (11.3%), suggesting SINEs were more active in the pig. However, not all SINE subfamilies were well represented in the pig genome. We found that SINE/tRNA was the most abundant class, including over 2.2 million copies with a total size of 325 Mbp, accounting for 12.4% of the genome (comparable with a previous report [20]), which is even more than the well known Alu elements derived from 7SL RNA in humans, estimated at 1 million copies and occupying 11.4% of the human genome. The length of SINE/tRNA elements ranged from 11 bp to 2,028 bp, but 77.8% of them ranged from 100–300 bp in length. The distribution of length revealed three peaks, the main peak located around 263 bp with smaller peaks at 106 bp and 55 bp. This is different from Alu in the human genome, which has a typical size of 300 bp (Figure S2 in Additional file 1).

Based on sequence similarity, the 2.2 million copies of pig SINE/tRNA can be divided into 47 groups; the consensus of each group was inferred based on multiple sequence alignments. The number of members in each group ranged from 1 to 887,807 and the top five biggest groups comprised more than 70% of the total SINE/tRNA sequences. In addition, 1.98 million or 88.5% TEs belong to tRNAglu-derived PRE1 class, which can be further divided into 14 subclasses. PRE1b, PRE1a, PRE1j, PRE1d2, PRE1k are the most abundant with more than 100,000 copies in each subclass. Only a few copies of *PRE1* can be found in cattle, and none of them can be detected in human and rodent genomes (Table S7 in Additional file 1). This result indicated a PRE1 expansion after speciation from the last common ancestor of pig and cattle. PRE1 was proposed to originate from the CHRS-S family [21], and it is widely distributed in *Phacochoerus aethiopicus* (warthog) and *Tayassu tajacu* (peccary), suggesting an expansion prior to speciation of the ancestral pig. The origin of PRE1 is estimated to be at least 43.2 Million years ago (Mya) [22]. Considering the divergence of cattle and pigs is estimated to have occurred around 54.1 Mya, the expansion of PRE1 can be narrowed down to between 43.2 and 54.1 Mya.

**Figure 2:**
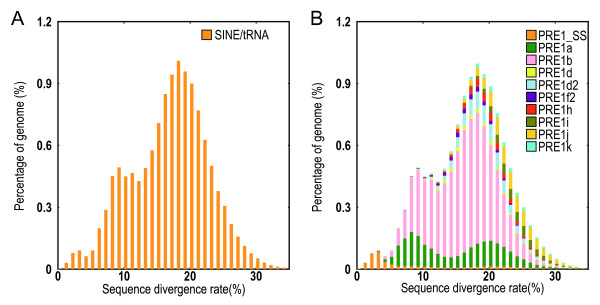
**Divergence distribution of SINE/tRNA in the pig genome. (A)** The divergence distribution of all SINE/tRNA, and **(B)** the divergence distribution of classified SINE/tRNA subfamilies.

Pair-wise comparison of transposable elements and their inferred consensus sequences were also investigated. The bimodal distribution of divergence with two peaks at ~ 20% and ~ 10% (Figure 2A) indicates that pig has undergone two rounds of SINE/tRNA expansion. The divergence of each of PRE1 subclass was also studied (Figure 2B), illustrating a similar pattern among all SINE/tRNA examined. It is clear that the peak at ~ 10% was almost exclusively due to recent expansions of PRE1a and PRE1b.

### Gene prediction and evolution

To understand the protein coding genes in the pig genome, homologous searching and transcription evidence-based prediction were performed. Protein sequences from closely related mammals and expressed sequence tags (ESTs) were used as query sequences to search for their counterparts in the assembly. We identified 20,326 genes. The average length of gene, exon and intron are approximately 29.4 Kb, 1.5 Kb and 3.5 Kb respectively, which is similar to cattle (33.6 Kb, 1.5 Kb and 3.9 Kb) and horse (32.3 Kb, 1.5 Kb and 3.7 Kb). Based on the reciprocal best BLAST alignment, 16,564, 17,475 and 16,923 orthologous groups were identified in pig/human, pig/cattle and pig/horse, respectively, and the distribution of sequence similarities of orthologs revealed that the pig is most closely related to cattle with an average amino acid identity of 85.9%, while sharing an average identity of 84.1% with human (Figure S 3).

**Figure 3:**
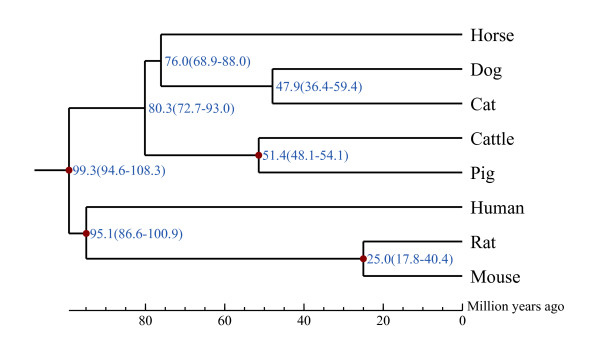
**Estimation of divergence time.** The time of divergence (with error range shown in parentheses) of pig and seven other mammals based on orthology relationships. Distances are shown in millions of years.

A TreeFam-based gene family analysis was conducted to study gene family evolution and estimate the divergence time of pig from other sequenced mammals. Gene sequences of human, horse, dog, cat, cattle, rat and mouse were used in this analysis (see Supplementary Text 3.2 in Additional file 1). We found that 18,814 or 92.6% of pig genes can be assigned into 9,360 gene families, at least with one other species. A total of 3,211 single copy gene orthologous groups were obtained, and sequences for each species were concatenated into a super gene to infer the phylogenetic tree (Figure 3). As expected, pig and cattle clustered together to represent Artiodactyla - both sharing a last common ancestor approximately 51.4 Mya.

To obtain better insight into the dynamic changes of evolution, gene family expansion and contraction were investigated by considering gene birth and death in an evolutionary framework [23]. By comparing the pig genome to cattle genome, which is its closest relative, we identified 27 expanded gene families (810 genes) while 17 gene families (169 genes) showed contraction. A typical example of a gene family expansion points to the functional categories including drug metabolism and synthesis of lipids, in which six copies of *Cyp2J2* were detected and originated from tandem duplication. A gene family related with somatotropin hormone function was contracted in the pig, which might underlie the smaller adult size of this inbred mini pig. Additionally, 240 orphan genes were identified with no counterpart in any other organisms under current criteria. They were clustered into 39 groups, representing pig-specific genes which may be due to gene gain or loss, or fast evolution, thus suggesting functional changes. Olfactory receptors and signaling-related genes were enriched in pig specific families (Table S11 in Additional file 1), suggesting fast evolution and/or unique regulation in the pig lineage. Genes of viral origin were also over represented in the pig-specific gene set due to the presence of porcine endogenous retroviruses (PERVs), which are known to be integrated into the pig genome.

A comparison of gene content between human and pig allows us to identify genes gained and lost during evolution (see Supplementary text 3.2 in Additional file 2). In total, 245 genes were identified as gained in pig (Table S12 in Additional file 2), and may be associated with the emergence of specific functions and physiology. For instance, the gene *WZSP010943* specifically existed in the pig genome compared to human, which encodes the interferon-induced transmembrane protein, and is linked with the proliferation, migration and invasion of glioma cells. It has also been proposed as a potential therapeutic target for gliomas [24]. In addition, our analysis identified 270 lost genes compared to human (Table S13 in Additional file 2); however, this may result from human gain or pig loss, as well as high sequence diversity, thus it is filtered under current criteria. For example, gene *LDHAL6A* was lost in the pig genome, but exclusively expressed in human testis, indicating human gain or pig loss events occurred after the divergence of these two species. Particular care should be taken if using pig as a model for studying human biology associated with these genes. We found that *PROZ*, the gene encoding protein Z (a member of blood proteins that leads to the formation of blood clots and thrombosis [25]), is lost in pig. The *CETP* gene is also lost and encodes the cholesteryl ester transfer protein - a plasma protein that facilitates the transport of cholesteryl esters and triglycerides between the lipoproteins. Heightening interest in *CETP* has been stimulated due to the discovery that *CETP* inhibitors are intended to reduce the risk of atherosclerosis by improving blood lipid levels, thereby benefiting patients with cardiovascular disease [26-28]. Pyridoxal phosphatase (encoded by the gene *PDXP*) is also lost (Table S13 in Additional file 2), and participates in vitamin B6 metabolism, acting as a catalyst in the hydrolysis of pyridoxal phosphate [29].

Pseudogenes are DNA sequences that resemble functional genes but are generally thought to have lost function, implicating a biological and evolutionary story behind the sequences. By mapping human protein sequences against the pig assembly, we detected 105 pseudogenes in pig (Table S14 in Additional file 2). Among these genes, some play an important role in organism development and physiological processes, including *UCP1AGR3CLDM6NMBRKCNK18GANC* and *CES2*. For example, Pseudogene *UCP1*, which was disrupted about 20 million years ago, provides an explanation for the lack of brown adipose tissue and poor thermoregulation in piglets [30]. Pseudogene *KCNK18* inactivates the potassium channel subfamily K member 18, which may help trigger pain centers in the brain and cause severe headaches [31,32]. Another pseudogene *GANC* inactivates the neutral alpha-glucosidase, a key enzyme involved in glycogen metabolism and associated with susceptibility to diabetes [33]. Interestingly, pseudogene *NMBR* was only found in the WZSP inbred-line, not in Bama miniature pig or large white pig, as confirmed by PCR validation. *NMBR* belongs to the GPCR family, and is a receptor for Neuromedin B (*NMB*), which is a mammalian homolog of amphibian bombesin. The NMB/NMBR pathway is involved in the regulation of a wide variety of physiological processes and behaviors, such as thermoregulation, cell growth, food intake and anxiety-related behavior [34]. Disruption of the neuromedin B receptor gene results in dysregulation of the pituitary-thyroid axis and partial resistance to diet-induced obesity, as well as a decrease in burying behavior through increasing levels of serotonin in the brain [35,36]. Lack of functional neuromedin-B receptor in WZSP may have functional implications for the unique physiology and behavior of WSZ inbred pigs. Here we provide the list of pseudogenes for further study by the research community.

Genes that have undergone positive selection can provide very useful pointers to the adaptation process during recent evolution. In this study, we identified 19 genes with evidence of positive selection (Table S15 in Additional file 2), including five DNA-repair and cell cycle control-related genes (*LSM10APLFTP53I13NEIL3CDKN3*), and five genes involved in organism development (*COG1CHGBGLIS2FECHSTK16*). One example of these positively selected genes is *CHGB*, which encodes chromogranin B, a neuroendocrine secretory granule protein that has an effect in reducing the availability of glucose and lowers the risk of cancer during aging [37]. Up-regulation of chromogranin B has been observed in dwarf mice and fasting control mice [38]. Therefore, the fast evolution of this gene might yield insights into dwarfism. Another gene under positive selection is *GLIS2*, which encodes the GLIS family zinc finger 2, which plays an essential role in kidney development and neurogenesis [38].

Although it is not clear how to make the connection between the unusual evolutionary history of a gene and the unique traits of pigs without additional functional experiments, the data set showed can be a valuable resource for further study. Most importantly, attention should be given to understanding the situation of target genes when using pig as an animal model for biomedical studies.

### Porcine endogenous retrovirus in the pig genome

Many patients with end-stage organ failure are dying from worldwide shortages of human organ donors. Xenotransplantation was proposed to be an efficient alternative, and as humans' closest relatives, nonhuman primates were originally considered to be the best organ donor. However, ethical restrictions, shortages and endangered status of some species, together with the risk of pathogen transmission from nonhuman primates to human, have impeded their application.

The similarities between humans and pigs at anatomical, physiological and genomic levels provide unique advantages for the use of pigs as a potential donor species for xenotransplantation [39]. Economic advantages and ethical considerations also promote their biomedical use. Furthermore, inbred WZSPs are particularly suitable for pig-to-human xenotransplantation [40], due to their physical size, as well as their inter-individual similarity. However, in addition to the immunological barriers, porcine endogenous retroviruses (PERVs) created an obstacle for safe transplantation. PERVs are ancient viral sequences integrated into the pig genome, and transmit vertically to the offspring, making them very difficult to eliminate. Since *in vitro* studies indicated that PERV released from porcine cells may infect human cells, the potential risks have sparked great concern over the use of pigs in xenotransplantation [41-46]. Therefore, it is of great importance to investigate PERVs in the pig genome.

**Figure 4:**
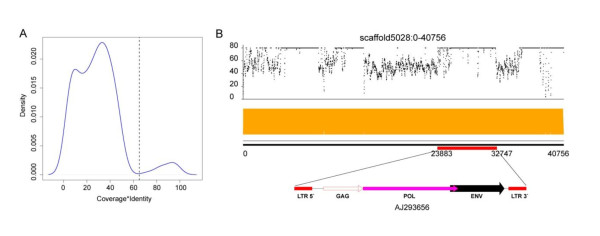
**PERVs in the WZSP genome. (A)** Distribution of similarity between virus protein and its counterpart in WZSP genome by considering factors of sequence identity and coverage. **(B)** Structure of whole PERV genome inserted into the pig genome. The upper panel shows the sequencing depth of the assembled scaffold. The middle panel visualizes the paired-end alignment of the scaffold providing evidence of correct assembly. The bottom panel shows the genome structure of PERV and its integration site in the scaffold.

A complete PERV genome includes *gagpol* and *env* genes, as well as 5' and 3' LTRs. The *gag* gene encodes a core protein which is a group-specific antigen; *pol* encodes an intergrase, reverse transcriptase and protease, which play critical roles in the virus life cycle; and *env* encodes envelope proteins that determine host tropism of the virus [47]. We screened the pig genome with the PERV proteins (*gagenvpol*) as well as genome sequences, including PERV-A, PERV-B, PERV-C and PERV-C/A downloaded from NCBI (Supplementary text 4 in Additional file 1). In total, we detected 182 copies of potential virus-derived genes in the assembly, and the product of coverage and identity between virus protein and pig encoding counterpart showed clear bimodal distribution (Figure 4A). Most of the virus-derived sequences were distantly related to queries, suggesting that many mutations were accumulated after the ancient integration event, whereas the remaining mutations were almost identical to virus sequences, indicating that those PERVs might still be active and replicating in the pig genome. Indeed, the polymorphic nature of PERV integration sites [48] within the pig genome suggests the independence of integration. When it comes to pig-to-human infection, more attention should be paid to active PERVs with full-length sequences.

When Filtered with identity and coverage > 80%, we identified eight virus-derived protein coding genes, including 5 *env*, 1 *gag* and 2 *pol* (Table S16 in Additional file 1). Among them, 3 *env*, 1 *gag* and 2 *pol* genes were from PERV-A, and 2 *env* genes from PERV-B. Only 1 *env* gene from PERV-A was intact, and no PERV-C derived genes could be found. In addition, we detected two copies of complete sequences of PERV integrated into the genome (Table S17 in Additional file 1). Figure 4B shows the evidence of the integration of a copy of PERV in the middle of the assembled scaffold, and the integrating points were well supported by paired-end reads.

A previous study suggested that PERVs might be removed by breeding or gene knockout techniques [47], and great effort has been made with the aim of eliminating PERV from the donor pig [49]. Since type C retroviruses like MuLV, BaEV, GALV and HERV were able to cause disorders in the host, such as leukemia, carcinoma or immunodeficiency, the highly maintained conservation of PERV-C [50] raises concerns of pig-to-human infection. The natural lack of PERV-C in the WZSP breed, together with the small amount of replication-competent PERV in its genome, might facilitate its application in pig-to-human xenotransplantation.

### Human druggable domain and disease associated orthologs in pig

The similarities between humans and pigs make pigs attractive as an animal model for biomedical studies. Pre-existing pig models of human diseases include a broad array of biomedical topics, such as heart physiology, reproductive function, skin physiology, brain function, transplantation, gut physiology and nutrition, tissue engineering, respiratory function and infectious disease models, to name a few (for a review see reference [2]). It is worth noting that pigs are the most promising animal model for atherosclerosis and cardiovascular disease. More specifically, the cardiovascular anatomy and hemodynamics of pigs are comparable to humans; they develop human-like spontaneous and diet-induced atherosclerotic lesions, and can experience sudden death when under stress [51-54]. Unlike small animal models, the progression of pig atherosclerosis can be generally quite slow, and both atherosclerotic plaque distribution and composition follows a pattern comparable to that of humans [55-57]. As miniature pigs, WZSPs are particularly suitable for biomedical research, due to their reasonable size and early maturity.

Understanding the pig counterparts of human drug targets is vital for pre-clinical drug screening, using pigs as a model organism. We downloaded the drug target gene information from DrugBank [58], and identified the counterparts in mouse, macaque and pig genomes. The DrugBank information showed that 1,624 druggable human genes have an ortholog in at least one of these three species: 1,616 are shared between human, mouse, macaque and pig, and 1,618 genes were identified in pig. The distribution of protein sequence identity between human and pig genes shows a peak at 95% (Figure S6 in Additional file 1). Compared to mouse and macaque, pig has specifically lost three drug target genes (*REG1APROZ* and *HSD3B2*), but retains *S100A12* and *GNLY*, which have been lost in the mouse lineage (Figure S7, in Additional file 1 Table S18 and Table S19 in Additional file 2). Comparing the differences between drug target genes in human and their counterparts in pig will provide vital information for biomedical studies. For example, *CYP3A4* is a hepatic microsomal P450, responsible for the oxidative metabolism of over 50% of clinically relevant drugs. Two post-translational modification sites [59] (Thr264 and Ser478) are responsible for phosphorylation and subsequent ubiquitin-dependent proteasomal degradation in human. Interestingly, amino acid substitutions were discovered in these two modification sites in pig (T264Q and S478T), such substitutions may make this protein more stable for oxidative biotransformation of various endo- and xenobiotics, and further biomedical studies focusing on the *CYP3A4* gene (using the pig as a model) should be aware of such modifications (Figure S8 in Additional file 1).

Amongst 247 coronary artery disease (CAD) related genes, only *ARMS2* and *CETP* were lost in our assembly (Table S20 in Additional file 2). The loss of *CETP* explains why its activity was undetectable in a previous study [60] while *ARMS2* is a primate-specific gene. We detected six copies of *Cyp2J2* that may have resulted from tandem duplication, and phylogenetic analysis reveals its expansion in the pig and mouse lineages (Figure S9 in Additional file 1). *Cyp2J2* is involved in various types of drug metabolism and synthesis of cholesterol, steroids and other lipids, and can help increase functional recovery of cardiomyocytes [61]. This tandem duplicated cluster may have similar or divergent functionality and/or regulation, and may underlie the differences seen in drug metabolism in the pig.

Though pig and human share most CAD genes, we also found a few differences. For instance, Fibrinogen alpha chain (*FGA*) and fibrinogen beta chain (*FGB*) both function in fibrin formation and platelet aggregation. The proper functions require proper cleavage of its protein sequences. Using the UniProtKB/Swiss-Prot database we found that the cleavage sites between R123 & D124 in *FGA*[62] and K152 & D153, K163 & D164 in *FGB*[63] are recognized by plasmin and break down fibrin clots. In the pig genome, we found amino acid substitutions in these cleavage sites (R123D in *FGA* and D153R, K163R in *FGB*). Such modifications may result in a different pattern of cleavage of these two proteins, thus suggesting potential physiological differences in fibrin clot degradation (Figure S10 in Additional file 1).

Another example is plasminogen activator inhibitor 2 (*PAI-2*), one drug target for tenecteplase (DrugBank ID: DB00031) and urokinase (DrugBank ID: DB00013). Three mutations (C5Y,C79G and C405N) were observed in the pig genome, and such substitutions may result in the loss of disulfide bonds (C5-C405 and C79-C161), which may prevent the polymerogenic conformation of PAI-2 [64] (Figure S11 in Additional file 1).

Clear genetic information will enhance the possibilities of creating useful WZSP models, and may lead to a better understanding of the molecular mechanisms underlying cardiovascular disease.

## Discussion

Our investigation on heterozygosis of the WZSP inbred diploids revealed an unexpectedly high rate of polymorphism maintained in certain genomic regions. Genomic regions with extremely low rates of heterozygosis (< 0.001%) account for 60% of the genome, indicating that of inbreeding has purged a large proportion of heterozygosity from the genome. However, the mechanism to explain why high heterozygosis was maintained during inbreeding remains unclear. Recessive lethality may be one reason, but it cannot be the explanation for all heterozygous regions, which account for more than 30% of the genome. Recombination and artificial selection during inbreeding might be other factors to consider. Sequencing of non-inbred WZSP as an out-group and the inbreeding pedigree will be interesting and should help us to understand how heterozygosis decreased or was maintained, and how chromosome structures reorganized during the inbreeding process from generation to generation.

Prediction and annotation of transposable elements in the pig genome showed over representation of tRNA derived TE, with approximately 2.2 million copies accounting for 12.4% of the genome. According to previous studies, SINE RNAs have been proven to impact gene expression and regulation [65-67]. The over representation of tRNA-derived SINEs in the pig genome may also play certain roles, not only in shaping the genome structure, but also in increasing the complexity of gene regulatory networks and population structure, which may have resulted from unequal SINE-SINE crossover by associating genes with new *cis*-elements. The evolution and influence of Alu in the human genome has been extensively studied, whereas the function of SINE/tRNA in the pig genome still needs to be explored in the future. Most of the TEs are proposed to have escaped from selection, thus the Suidae-specific expansion of the TRE1 class is a valuable resource to understand the evolutionary history of Suidae, as well as the process of domestication and selective breeding. Further, population scale and functional genomics will deepen our understanding of its role in shaping the pig genome.

Based on our analysis of porcine endogenous retrovirus, we detected a limited number of active PERVs and a natural lack of PERV-C in the WZSP genome, which lowers the risk of pig-to-human infection during xenotransplantation - highlighting the use of these pigs as potential organ donors. A detailed investigation on gene evolution in the pig, as well as research on the pig counterparts of human druggable domain and disease related genes, revealed that pigs strongly resemble human beings, but also demonstrates that attention should be paid to the differences between human and pig when taking pig as an animal model.

## Methods

The genome was sequenced on the Illumina HiSeq™ 2000 platform (Illumina, San Diego, CA, USA). The sequenced individual male WZSP was from the Institute of Animal Science of CAAS, Beijing, China. The genome was assembled using SOAPdenovo. See Supplementary Information for data analysis and additional details.

## Availability of supporting data

The WZSP whole genome shotgun projects have been deposited at DDBJ/EMBL/GenBank under the accession number of AJKK00000000. The version described in this paper is the first version, AJKK01000000. All short read data have been deposited into the Short Read Archive under accession number of SRA051254. Genome assemblies and annotation files are also available from the *GigaScience* database [11].

## Abbreviations

bp: base pair; CAAS: Chinese Academy of Agriculture Science; CAD: Coronary artery disease; CDS: Coding sequence; EST: Express sequence tag; FGA: Fibrinogen alpha chain; FGB: Fibrinogen beta chain; LINE: Long interspersed element; LTR: Long terminal repeat; Mya: Million years ago; NMB: Neuromedin B; NMR: Naked mole rat; PAI-2: Plasminogen activator inhibitor 2; PERV: Porcine endogenous retroviruses; SINE; Short interspersed elements; SNP: Single nucleotide polymorphism; TE; Transposable element; WZSP; Wuzhishan pig.

## Competing interests

The authors declare no competing interests.

## Author contributions

Jun Wang, Jian Wang, SL, SF, HY and LL conceived the study. YD and KL supervised the study. YM, HL and CL prepared samples. JW and GW performed the DNA library construction and sequencing. XJ, LY, YC, ZX, DF, LR, LM, ZH, LH, XS, YF, WZ, YZ, YL, HL and CL performed he genome assembly, gene annotation, gene evolution and animal model study. XF, ZH, LH, YM, YL, LM, and YZ discussed the data. XF, ZH, LH and YZ and YL wrote the manuscript with significant contributions from all other co-authors. All authors read and approved the final manuscript.

## Supplementary Material

Additional file 1 The sequence and analysis of a Chinese pig genome method. Click here for file

Additional file 2 Supplementary tables. Click here for file
