# Supplementary material for: The sequence and analysis of a Chinese pig genome
Source: Gigascience. 2012 Nov 15;1:16. doi: 10.1186/2047-217X-1-16 (PMC3626506; doi:10.1186/2047-217X-1-16)
Supplement: Additional file 1 — The sequence and analysis of a Chinese pig genome method. [file 2047-217X-1-16-S1.doc]

**Supplementary Information:**

**The sequence and analysis of a Chinese inbred pig genome**

1 Genome sequencing and assembly [2](#__RefHeading___Toc325639543)

1.1 Genome sequencing [2](#__RefHeading___Toc325639544)

1.2 Estimation of genome size using k-mer [2](#__RefHeading___Toc325639545)

1.3 Genome assembly [3](#__RefHeading___Toc325639546)

2 Genomic feature [4](#__RefHeading___Toc325639547)

2.1 Heterozygosis of the diploid [4](#__RefHeading___Toc325639548)

2.2. Transposable elements in pig genome [6](#__RefHeading___Toc325639549)

2.3 Gene annotation [8](#__RefHeading___Toc325639550)

2.4 Gene functional annotation [9](#__RefHeading___Toc325639551)

2.5 Orthology relationship [10](#__RefHeading___Toc325639552)

3 Gene evolution [11](#__RefHeading___Toc325639553)

3.1 Gene family clusters [11](#__RefHeading___Toc325639554)

3.2 Phylogenetic analysis [11](#__RefHeading___Toc325639555)

3.3 Analyses of gene gain and loss [13](#__RefHeading___Toc325639556)

3.4 Pseudogene identification in pig genome [13](#__RefHeading___Toc325639557)

3.5 Positively selected genes [14](#__RefHeading___Toc325639558)

4 Porcine endogenous retrovirus (PERVs) analysis [14](#__RefHeading___Toc325639559)

5 Druggable domains and human disease related genes [15](#__RefHeading___Toc325639560)

6 Supplementary references [19](#__RefHeading___Toc325639561)

# 1 Genome sequencing and assembly

## 1.1 Genome sequencing

We applied a whole genome shotgun strategy and next-generation sequencing technologies using Illumina HiSeq 2000 to sequence the genome of Wuzhishan pig (WZSP). DNA was extracted from an individual adult male WZSP. In order to lower the risk of non-randomness, we constructed 16 paired-end libraries by sequencing 25 lanes, with insert sizes of about 170 base pairs (bp), 350 bp, 500 bp, 800 bp, 2 kbp, 5 kbp, 10 kbp and 20 kbp. In total, we generated about 340.53G of sequence, and 210.79G (78x coverage) was retained for assembly after filtering out low quality and duplicated reads.

**Supplementary Table 1. Data production.**

| **Pair-end libraries** | **Insert size** | **Total data (G)** | **Read length** | **Sequence coverage* (X)** | **Physical coverage (X)** |
| --- | --- | --- | --- | --- | --- |
| Illumina reads | 170bp | 38.15 | 100 | 14.13 | 25.29 |
|  | 350bp | 42.09 | 100 | 15.59 | 57.43 |
|  | 500bp  800bp | 37.77 | 100 | 13.99 | 73.62 |
|  |  | 30.93 | 100 | 11.45 | 96.46 |
|  | 2kb  5kb | 29.34 | 49 | 10.87 | 443.57 |
|  |  | 16.80 | 49 | 6.22 | 634.77 |
|  | 10Kb | 8.79 | 49 | 3.26 | 664.64 |
|  | 20kb | 6.93 | 49 | 2.57 | 1,047.44 |
| Total |  | 210.79 |  | 78.07 |  |

*The genome size was estimated at 2.7 Gbp

## 1.2 Estimation of genome size using k-mer

A k-mer refers to an artificial sequence division of K nucleotides iteratively from sequencing reads. A raw sequence read with L bp contains (L-K+1) k-mers if the length of each k-mer is K bp. The frequency of each k-mer can be calculated from the genome sequence reads. Typically, k-mer frequencies plotted against the sequence depth gradient follow a Poisson distribution in any given dataset; whereas sequencing errors may lead to a higher representation of low frequencies. The genome size, G, can be calculate from the formula G=K_num/K_depth, where the K_num is the total number of k-mer, and K_depth denotes the frequency occurring more frequently than the other frequencies. In this work, K was 17, K_num is 47,274,868,819 and K_depth was 19; the WZSP genome size was therefore estimated to be 2.5 Gbp.


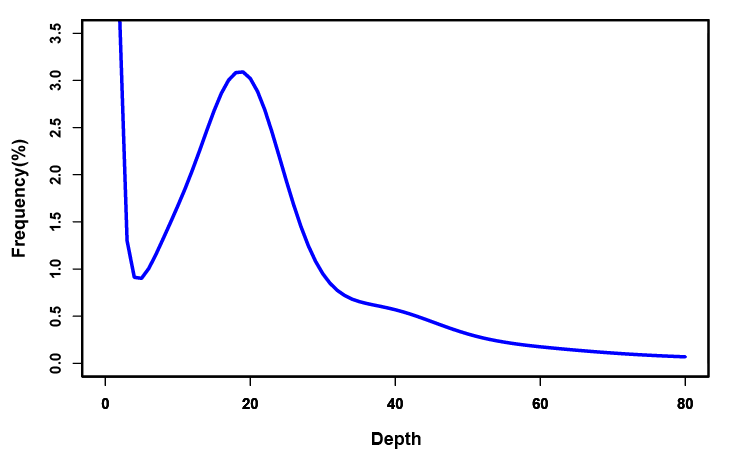


**Supplementary Fig. 1. 17-kmer estimation of genome size.** The genome size of pig was estimated to be 2.5G based on reads from short insert size libraries.

## 1.3 Genome assembly

The genome was de novo assembled by SOAPdenovo[^1^](#_ENREF_1) (http://soap.genomics.org.cn), a novel short-read assembly method that employs the *de Bruijn* graph algorithm in order to both simplify the task of assembly and reduce computational complexity. Firstly, reads with low quality or with potential sequencing errors were removed or corrected by k-mer frequency based methodology. After these quality control and filtering steps, a total of 211 Gbp (or 78X) data were retained for assembly. SOAPdenovo first constructs the de Bruijn graph by splitting the reads from short insert size libraries (170-800 bp) into 41-mers and then merging the 41-mers; contigs which exhibit unambiguous connections in de Bruijn graphs are then collected. All reads were aligned onto the contigs for scaffold building using the paired-end information. This paired-end information was subsequently used to link contigs into scaffolds, step by step, from short insert sizes to long insert sizes. About 149G (or 55X) data were used to build contigs, while all high quality data were used to build scaffolds. Some intra-scaffold gaps were filled by local assembly using the reads in a read-pair, where one end uniquely aligned to a contig while the other end was located within the gap. The final total contig size and N50 were 2.57 Gbp and 23.5 Kbp, respectively. The total scaffold size and N50 were 2.63 Gbp and 5.4 Mbp, respectively (Supplementary Table 2). To assess assembly quality, high quality reads that satisfied our filtering criteria of short insert size libraries were aligned onto the assembly using BWA[^2^](#_ENREF_2) with default parameters. A total of 98.08% reads could be mapped and they covered 97.37% of the assembly, excluding gaps. This result suggests that nearly the entire pig genome was represented in our assembly.

**Supplementary Table 2. Statistics of the assembled genome.**

|  | **Contig** | | **Scaffold** | |
| --- | --- | --- | --- | --- |
|  | **Length** | **Number** | **Length** | **Number** |
| N90 | 3,491 | 126,851 | 292,864 | 790 |
| N80 | 8,928 | 83,474 | 1,334,634 | 431 |
| N70 | 13,630 | 60,307 | 2,425,618 | 285 |
| N60 | 18,410 | 44,096 | 4,036,142 | 199 |
| N50 | 23,535 | 31,739 | 5,432,118 | 143 |
| Total Size | 2,572,895,595 |  | 2,636,530,514 |  |
| Longest | 229,558 |  | 21,409,172 |  |
| Total Number(>100bp) |  | 1,306,720 |  | 1,138,136 |
| Total Number(>2kb) |  | 145,786 |  | 5,843 |

# 2 Genomic feature

## 2.1 Heterozygosis of the diploid

We aligned the reads to the assembly using BWA[^2^](#_ENREF_2) with parameters -t 1 -I. Since the alignment results were stored in BAM/SAM format, SAMtools, which is based on the Bayesian model, was selected for variation analysis[^3^](#_ENREF_3). After sorting alignments by leftmost coordinates and removing potential PCR duplicates, we used SAMtools pileup to call SNPs and short InDels.

We rejected SNPs and InDels within reads with depth either much lower or much higher than expected, since large copy number variation might lead to miscalling of SNPs. The sequencing depth ranged from 10 to 80, and the upper limit was about twice the sequencing depth. The SNP-miscalling caused by alignment around short InDels and low-quality sequences were removed. We applied samtools.pl varFilter as the filtered tool, which can be found in the SAMtools package, with parameters -Q 20 -q 20 -d 10 -D 80 -S 20 -i 20 -N 5 -l 5 -W 5 -N 1.

**Supplementary Table 3. GO enrichment of the top 1000 highest heterozygous genes.**

| **GO_ID** | **GO_Term** | **GO Class** | **GO level** | **#Gene** | **Adjusted**  **P-value** |
| --- | --- | --- | --- | --- | --- |
| GO:0004984 | olfactory receptor activity | MF | 7 | 274 | 8.26E-220 |
| GO:0007186 | G-protein coupled receptor protein signaling pathway | BP | 5 | 317 | 1.34E-177 |
| GO:0004930 | G-protein coupled receptor activity | MF | 6 | 284 | 1.63E-168 |
| GO:0007166 | cell surface receptor linked signaling pathway | BP | 4 | 319 | 9.41E-167 |
| GO:0004888 | transmembrane receptor activity | MF | 5 | 289 | 2.85E-146 |
| GO:0004872 | receptor activity | MF | 4 | 293 | 4.58E-134 |
| GO:0004871 | signal transducer activity | MF | 3 | 294 | 9.87E-118 |
| GO:0016021 | integral to membrane | CC | 5 | 324 | 1.27E-104 |
| GO:0031224 | intrinsic to membrane | CC | 4 | 325 | 1.18E-102 |
| GO:0007165 | signal transduction | BP | 3 | 326 | 2.50E-102 |
| GO:0023052 | signaling | BP | 2 | 327 | 6.40E-102 |
| GO:0051716 | cellular response to stimulus | BP | 3 | 328 | 2.13E-97 |
| GO:0050896 | response to stimulus | BP | 2 | 347 | 1.04E-94 |
| GO:0044425 | membrane part | CC | 3 | 332 | 1.94E-91 |
| GO:0050794 | regulation of cellular process | BP | 3 | 359 | 3.94E-65 |
| GO:0065007 | biological regulation | BP | 2 | 363 | 3.52E-63 |
| GO:0016020 | membrane | CC | 3 | 352 | 2.20E-48 |
| GO:0044464 | cell part | CC | 2 | 448 | 9.93E-18 |
| GO:0009987 | cellular process | BP | 2 | 453 | 1.42E-15 |
| GO:0004867 | serine-type endopeptidase inhibitor activity | MF | 6 | 12 | 0.0013545 |
| GO:0006952 | defense response | BP | 4 | 13 | 0.00342747 |
| GO:0030414 | peptidase inhibitor activity | MF | 4 | 14 | 0.0050975 |
| GO:0003735 | structural constituent of ribosome | MF | 3 | 24 | 0.02262732 |
| GO:0005840 | ribosome | CC | 4 | 24 | 0.02389858 |

**Supplementary Table 4. Heterozygosis distribution in Pig, NMR and human genome.**

|  | **Region** | **Size** | **Effective size** | **#SNP** | **%SNP** | **#InDel** | **%Polymorphism** |
| --- | --- | --- | --- | --- | --- | --- | --- |
| Pig | intergenic | 2,041,398,582 | 1,800,550,004 | 2,161,559 | 0.12 | 308,185 | 0.14 |
|  | gene | 595,103,379 | 567,339,278 | 635,888 | 0.11 | 84,082 | 0.13 |
|  | intron | 563,889,444 | 538,653,921 | 616,175 | 0.11 | 83,734 | 0.13 |
|  | cds | 31,213,935 | 28,685,357 | 19,713 | 0.07 | 348 | 0.07 |
|  | total | 2,636,501,961 | 2,367,889,282 | 2,797,447 | 0.12 | 392,267 | 0.13 |
| Human | intergenic | 1,917,369,926 | 1,917,369,926 | 2,030,248 | 0.11 | 92,593 | 0.11 |
|  | gene | 1,190,307,347 | 1,190,307,347 | 1,043,849 | 0.09 | 42,606 | 0.09 |
|  | intron | 1,121,178,122 | 1,121,178,122 | 1,026,073 | 0.09 | 42,541 | 0.10 |
|  | cds | 69,129,225 | 69,129,225 | 17,776 | 0.03 | 65 | 0.03 |
|  | total | 3,107,677,273 | 3,107,677,273 | 3,074,097 | 0.10 | 135,199 | 0.10 |
| NMR | intergenic | 1,942,431,872 | 1,729,989,151 | 1,312,642 | 0.08 | 430,083 | 0.10 |
|  | gene | 722,334,413 | 674,361,764 | 382,165 | 0.06 | 105,540 | 0.07 |
|  | intron | 689,877,137 | 642,400,325 | 366,466 | 0.06 | 104,504 | 0.07 |
|  | cds | 32,457,276 | 31,961,439 | 15,699 | 0.05 | 1,036 | 0.05 |
|  | total | 2,664,766,285 | 2,404,350,915 | 1,694,807 | 0.07 | 535,623 | 0.09 |

## 2.2. Transposable elements in pig genome

We employed the software Tandem Repeats Finder (TRF)[^4^](#_ENREF_4) to identify tandem repeats.

RepeatMasker[^5^](#_ENREF_5) and RepeatProteinMask were used to identify and classify transposable elements (TEs) by aligning the pig genome sequences against a library of known repeats, Repbase, with default parameters. To better compare with other mammals, we used the same pipeline and parameters to re-run the repeat annotation in cattle, horse, human, mouse and rat genomes as shown in Supplementary Table 5.

**Supplementary Table 5. TE comparison of pig** and other mammalian genomes.

|  | **pig** | | **cattle** | | **horse** | | **human** | | **mouse** | | **rat** | |
| --- | --- | --- | --- | --- | --- | --- | --- | --- | --- | --- | --- | --- |
|  | **Length (Mp)** | **% genome** | **Length (Mp)** | **% genome** | **Length (Mp)** | **% genome** | **Length (Mp)** | **% genome** | **Length (Mp)** | **% genome** | **Length (Mp)** | **% genome** |
| DNA | 59 | 2.2 | 63 | 2.2 | 82.9 | 3.4 | 102.4 | 3.3 | 63.7 | 2.3 | 66.8 | 2.5 |
| LINE | 477 | 18.1 | 760 | 26.0 | 479.4 | 19.4 | 543.0 | 17.5 | 495.3 | 18.2 | 529.5 | 19.5 |
| LTR | 120 | 4.5 | 136 | 4.7 | 167.3 | 6.8 | 257.2 | 8.3 | 283.9 | 10.4 | 220.8 | 8.1 |
| SINE | 360 | 13.6 | 282 | 9.7 | 60.8 | 2.5 | 349.4 | 11.3 | 166.7 | 6.1 | 144.6 | 5.3 |
| Other | 0 | 0.0 | 0 | 0.0 | 0.0 | 0.0 | 26.4 | 0.9 | 7.6 | 0.3 | 6.8 | 0.2 |
| Unknown | 0.7 | 0.0 | 0.8 | 0.0 | 1.6 | 0.1 | 4.8 | 0.2 | 43.7 | 1.6 | 50.7 | 1.9 |
| Total | 1,009 | 38.3 | 1,211 | 41.5 | 789.5 | 31.9 | 1257.7 | 40.5 | 1017.0 | 37.4 | 978.0 | 36.0 |

**Supplementary Table 6. TE statistics in six** mammalian genomes.

| **Species** | **pig** | | **cattle** | | **horse** | | **human** | | **mouse** | | **rat** | |
| --- | --- | --- | --- | --- | --- | --- | --- | --- | --- | --- | --- | --- |
| **Class** | **copy** | **%genome** | **copy** | **%genome** | **copy** | **%genome** | **copy** | **%genome** | **copy** | **%genome** | **copy** | **%genome** |
| SINE/tRNA | 2,237,379 | 12.36 | 256,750 | 1.53 | 8,498 | 0.11 | 909 | 0.00 | 7,788 | 0.02 | 8,354 | 0.02 |
| LINE/L1 | 1,229,038 | 16.80 | 870,506 | 11.90 | 725,846 | 16.54 | 831,144 | 18.35 | 818,384 | 17.77 | 776,312 | 19.01 |
| LTR/ERVL | 247,048 | 2.70 | 251,354 | 2.32 | 352,081 | 4.56 | 488,118 | 6.00 | 472,810 | 4.64 | 381,787 | 3.71 |
| SINE/MIR | 219,114 | 1.18 | 313,297 | 1.28 | 386,401 | 2.33 | 294,277 | 1.64 | 10,628 | 0.05 | 11,165 | 0.05 |
| DNA/hAT | 218,403 | 1.37 | 238,791 | 1.31 | 304,949 | 2.20 | 285,124 | 1.91 | 257,382 | 0.99 | 267,001 | 1.00 |
| LTR/ERV1 | 136,150 | 1.56 | 256,107 | 1.87 | 115,451 | 1.88 | 176,672 | 3.14 | 112,942 | 1.10 | 156,084 | 1.05 |
| LINE/L2 | 133,671 | 1.13 | 144,583 | 1.02 | 280,798 | 2.49 | 205,917 | 1.85 | 56,857 | 0.28 | 53,478 | 0.27 |
| DNA/TcMar | 64,351 | 0.56 | 74,003 | 0.60 | 86,577 | 0.89 | 130,815 | 1.45 | 65,632 | 0.29 | 68,621 | 0.29 |
| DNA/En-Spm | 42,387 | 0.12 | 48,034 | 0.11 | 23,695 | 0.07 | 67,973 | 0.22 | 171,360 | 0.48 | 185,309 | 0.52 |
| LTR/ERVK | 31,466 | 0.18 | 20,691 | 0.39 | 16,794 | 0.11 | 28,213 | 0.37 | 278,629 | 4.40 | 230,738 | 3.00 |
| LTR/Gypsy | 22,311 | 0.09 | 22,546 | 0.08 | 33,709 | 0.19 | 20,855 | 0.11 | 57,386 | 0.17 | 68,499 | 0.20 |
| LINE/CR1 | 19,258 | 0.11 | 12,984 | 0.08 | 31,770 | 0.24 | 13,527 | 0.12 | 1,120 | 0.01 | 1,274 | 0.01 |
| DNA/Sola | 17,713 | 0.06 | 16,908 | 0.06 | 10,599 | 0.05 | 25,407 | 0.10 | 119,518 | 0.45 | 140,749 | 0.56 |
| DNA/Maverick | 13,760 | 0.04 | 10,195 | 0.02 | 8,566 | 0.03 | 16,763 | 0.04 | 59,360 | 0.16 | 66,515 | 0.18 |
| DNA/Ginger | 13,296 | 0.04 | 0 | 0.00 | 5,493 | 0.02 | 0 | 0.00 | 0 | 0.00 | 0 | 0.00 |
| LINE/RTE | 9,205 | 0.04 | 610,690 | 13.05 | 9,761 | 0.09 | 4,216 | 0.03 | 2,673 | 0.01 | 2,907 | 0.01 |
| DNA/Helitron | 6,310 | 0.02 | 8,384 | 0.03 | 6,112 | 0.03 | 3,193 | 0.01 | 7,312 | 0.02 | 7,846 | 0.02 |
| DNA/Chapaev | 5,746 | 0.02 | 8,198 | 0.02 | 3,752 | 0.01 | 9,730 | 0.03 | 15,357 | 0.04 | 14,350 | 0.04 |
| DNA/MuDR | 4,668 | 0.02 | 6,994 | 0.02 | 4,492 | 0.01 | 13,428 | 0.08 | 18,119 | 0.06 | 17,820 | 0.06 |
| DNA/P | 4,541 | 0.01 | 7,768 | 0.02 | 2,831 | 0.01 | 9982 | 0.04 | 15,392 | 0.05 | 13,407 | 0.04 |

**Supplementary Fig. 2. Length distribution of SINE/tRNA in pig and Alu in human.**

**Supplementary Table 7. SINE/tRNA copy number in six mammalian genomes.**

| **Type** | **Copy_Numbers** | | | | | |
| --- | --- | --- | --- | --- | --- | --- |
| **Species** | **pig** | **cattle** | **horse** | **human** | **mouse** | **rat** |
| PRE1b | 887,807 | 0 | 0 | 0 | 0 | 0 |
| PRE1a | 296,550 | 0 | 0 | 0 | 0 | 0 |
| PRE1j | 194,174 | 7 | 0 | 0 | 0 | 0 |
| PRE1d2 | 148,625 | 0 | 0 | 0 | 0 | 0 |
| PRE1k | 108,027 | 2 | 0 | 0 | 1 | 0 |
| PRE1i | 88,378 | 1 | 0 | 0 | 0 | 0 |
| PRE1_SS | 80,786 | 0 | 0 | 0 | 0 | 0 |
| PRE1h | 72,730 | 5 | 0 | 0 | 0 | 1 |
| PRE1f2 | 53,320 | 2 | 0 | 0 | 0 | 0 |
| PRE1d | 50,269 | 0 | 0 | 0 | 0 | 0 |
| SINE1C_SS | 40,477 | 0 | 0 | 0 | 0 | 0 |
| SINE1B_SS | 37,000 | 0 | 0 | 0 | 0 | 0 |
| SINE1D_SS | 30,153 | 0 | 0 | 0 | 0 | 0 |
| SINE1A_SS | 29,208 | 5 | 0 | 0 | 0 | 0 |
| CHR-1 | 28,782 | 147 | 0 | 0 | 0 | 0 |
| SINE1_SS | 22,073 | 6 | 0 | 0 | 0 | 0 |
| Pre0_SS | 20,879 | 0 | 0 | 0 | 0 | 0 |
| PRE1g | 18,351 | 16 | 0 | 0 | 0 | 0 |
| SINE2-1_SSc | 9,804 | 0 | 22 | 0 | 0 | 0 |
| PRE1f | 7,077 | 18 | 0 | 0 | 0 | 0 |
| PRE1c | 6,953 | 3 | 0 | 0 | 0 | 0 |
| PRE1e | 4,658 | 0 | 0 | 0 | 0 | 0 |
| SUSINE2 | 1,112 | 0 | 0 | 0 | 0 | 0 |

## 2.3 Gene annotation

To predict protein-coding genes, we applied a homology-based method, using genes of human and cow as references. Human and cow proteins were downloaded from Ensembl (release 64) and mapped onto the genome using TblastN[^6^](#_ENREF_6). Homologous genome sequences were then aligned against the matching proteins using Genewise[^7^](#_ENREF_7) to define gene models. In total, 20,286 genes were identified in the pig genome. The EST sequences of the *Sus scrofa* from NCBI were also aligned against the genome using BLAT to generate spliced alignments. After filtering the overlaps, these spliced alignments were linked together using PASA[^8^](#_ENREF_8). Genes that had EST support and correct structure but were lost in homology-based prediction were added into the gene set. Finally, we obtained a reference gene set that contained 20,326 genes.

**Supplementary Table 8**. Statistics of predicted protein-coding genes.

| **Species** | **Gene set number** | **Single exon gene** | **%** | **Average transcript length (bp)** | **Average ORF length (bp)** | **Average exons per gene** | **Average exon length (bp)** | **Average intron length (bp)** |
| --- | --- | --- | --- | --- | --- | --- | --- | --- |
| Pig | 20,326 | 2,745 | 13.50 | 29,386 | 1,537 | 8.95 | 172 | 3,505 |
| Human | 21,642 | 2,449 | 11.32 | 48,472 | 1,574 | 9.25 | 170 | 5,681 |
| Cattle | 19,970 | 2,459 | 12.31 | 35,523 | 1,599 | 9.59 | 167 | 3,949 |
| Horse | 20,383 | 3,323 | 16.30 | 32,268 | 1,532 | 9.28 | 165 | 3,713 |

## 2.4 Gene functional annotation

We aligned the pig reference genes to proteins annotated in SwissProt and TrEMBL[^9^](#_ENREF_9) databases using BlastP, so as to obtain functional annotation of pig genes based on the best match derived from the alignments. InterPro[^10^](#_ENREF_10) was used to annotate motifs and domains by searching against publicly available databases, including Pfam, PRINTS, PROSITE, ProDom, and SMART. The descriptions of gene products, including Gene Ontology[^11^](#_ENREF_11), were retrieved from InterPro. We also mapped the pig reference genes to KEGG[^12^](#_ENREF_12) pathway maps by searching KEGG databases and finding the best hit for each gene.

**Supplementary Table 9. Functional classification of pig** genes with various methods.

|  | | **Number** | **Percent (%)** |
| --- | --- | --- | --- |
| Total | | 20,326 | 100.00 |
| Annotated | SwissProt | 19,244 | 94.68 |
|  | TrEMBL | 19,312 | 95.01 |
|  | KEGG | 15,209 | 74.83 |
|  | InterPro | 16,960 | 83.44 |
|  | GO | 14,372 | 70.71 |
| Unannotated | | 742 | 3.65 |

## 2.5 Orthology relationship

To determine the orthology relationship between WZSP and other mammalian proteins, nucleotide and protein data for other mammals (human, horse and cattle) were downloaded from the Ensembl database (release 64). The longest transcripts were chosen to represent genes with alternative splicing variants. We then subjected all the proteins to BlastP analysis with the similarity cutoff threshold of e=1e-5. With the pig protein set used as a reference, we found the best hit for each pig protein in other species, with the criteria that more than 30% of the aligned sequence showed an identity above 30%. Reciprocal best-match pairs were defined as orthologues. Statistics of pig and other mammalian orthologues is shown in Supplementary Table 10.

**Supplementary Table 10**. Orthologous relationship between pig and other mammals.

|  | **Orthologue counts** |
| --- | --- |
| pig:cattle | 17,475 |
| pig:horse | 16,923 |
| pig:human | 16,564 |

**Supplementary Fig. 3. Sequence identity of pig proteins in comparison with human, horse and cattle proteins.**

#

# 3 Gene evolution

## 3.1 Gene family clusters

A gene family denotes a set of similar genes that descended from a single original gene in the last common ancestor of considered species. In this study, we used the TreeFam methodology[^13^](#_ENREF_13) to define gene families, using seven mammals (human, horse, dog, cat, cattle, rat and mouse) as reference species. We carried out the same pipeline and parameters that we used in our previously published work.

## 3.2 Phylogenetic analysis

We constructed a phylogenetic tree of pig and several other sequenced mammalian genomes (human, horse, dog, cat, cattle, rat and mouse) using the same pipeline and parameters that we used in our previously published work.

**Supplementary Fig. 4. Expansion and contraction of gene families.** Numbers designate the number of gene families that have expanded (green) and contracted (red) since the split from the common ancestor. The most recent common ancestor (MCRA) has 10,833 gene families

**Supplementary Table 11**. GO enrichment of pig specific gene families

| **GO ID** | **GO Term** | **GO Class** | **GO level** | **#Gene** | **Adjusted**  **P-value** |
| --- | --- | --- | --- | --- | --- |
| GO:0004984 | olfactory receptor activity | MF | 7 | 136 | 3.63E-273 |
| GO:0004930 | G-protein coupled receptor activity | MF | 6 | 140 | 1.63E-220 |
| GO:0004888 | transmembrane receptor activity | MF | 5 | 141 | 7.08E-195 |
| GO:0016021 | integral to membrane | CC | 5 | 145 | 1.56E-130 |
| GO:0050794 | regulation of cellular process | BP | 3 | 147 | 1.30E-80 |
| GO:0016020 | membrane | CC | 3 | 149 | 4.77E-74 |
| GO:0044464 | cell part | CC | 2 | 155 | 2.08E-29 |
| GO:0009987 | cellular process | BP | 2 | 158 | 2.34E-29 |
| GO:0005581 | collagen | CC | 4 | 3 | 0.000557889 |
| GO:0005201 | extracellular matrix structural constituent | MF | 3 | 3 | 0.000707641 |
| GO:0004966 | galanin receptor activity | MF | 6 | 2 | 0.001368084 |
| GO:0019068 | virion assembly | BP | 4 | 2 | 0.001368084 |
| GO:0044423 | virion part | CC | 2 | 2 | 0.002296843 |
| GO:0004522 | pancreatic ribonuclease activity | MF | 9 | 2 | 0.022038304 |
| GO:0007156 | homophilic cell adhesion | BP | 5 | 4 | 0.022325738 |
| GO:0016032 | viral reproduction | BP | 2 | 3 | 0.023396452 |
| GO:0019031 | viral envelope | CC | 3 | 1 | 0.023438468 |
| GO:0046080 | dUTP metabolic process | BP | 9 | 1 | 0.043233421 |

## 3.3 Analyses of gene gain and loss

Since the orthology relationship showed synteny information at the protein level, it could be used to analyze gene gain and loss between human and pig. Within the protein synteny blocks, if a human protein had no pig counterpart, excluding false positive predictions that could be caused by annotation or genome assembly (gap > 5%), this protein could be identified as either being lost in the pig lineage or gained in the human lineage. Using pig proteins as a reference to generate the orthology relationship, we applied a similar procedure to identify genes gained in the pig lineage compared to the human lineage (Table S12-S13).

## 3.4 Pseudogene identification in pig genome

We aligned human proteins onto to the pig genome located in synteny blocks to call homologues. Since frameshift and premature termination events might occur in homologous regions, we manually examined genomic and transcriptomic reads mapping quality of frameshift and premature termination loci. Cases with high mapping quality, excluding any SNPs or InDels, were inferred as mutations, which in turn were identified as pseudogenes. Human proteins containing alternative splice variants were used to call homologues in identified WZSP pseudogene loci and we found that some alternative splice variants could stride over the mutation sites (Table S14).

## 3.5 Positively selected genes

The PAML branch-site test of positive selection[^16-19^](#_ENREF_16) was used to detect positively selected genes along the pig branch. We chose null model with fix_omega = 1 and omega = 1, alternative model with fix_omega = 0 and omega = 1.5. P-values were computed using the X^2^ statistic adjusted by the False Discovery Rate (FDR) method to allow for multiple testing. We further excluded positively selected genes that may be falsely obtained by sequence error or annotation error (Table S15).

# 4 Porcine endogenous retrovirus (PERVs) analysis

The intact genome sequences of known PERVs were mapped onto the WZSP genomes with BLAT to detect full-length endogenous retrovirus insertions defined as identity > 80% and coverage > 80%. In the following step, PERV proteins were mapped onto the assembly using TblastN, with an e-value cutoff of 1e-7. We then called on a homology-based prediction pipeline to analysis the putative PERV elements within the pig genome using GeneWise. Putative PERV peptides were also mapped onto the GenBank non-redundant (nr) database in a reciprocal TblastN search. We filtered out those sequences that matched to viral cloning vectors, as well as those non-specific matches to host loci. The remaining sequences were considered as PERV element sequences if the best matches are viral proteins.

**Supplementary Table** **16.** Copy number of PERV derived genes in pig genome

| **subtype** | **Virus Protein** | **Copy Number** |
| --- | --- | --- |
| PERV-A | env | 3 |
| PERV-A | gag | 1 |
| PERV-A | pol | 2 |
| PERV-B | env | 2 |
| Total |  | 8 |

**Supplementary Table 17. Location of PERV genome integrated into the pig genome**

| **Host** | **Virus_Genome** | **Type** | **Length** | **Chromosome** | **Strand** | **Start** | **End** |
| --- | --- | --- | --- | --- | --- | --- | --- |
| Pig | AJ293656 | PERV-A | 8,918 | scaffold640 | + | 1,378,451 | 1,386,833 |
| Pig | AJ293656 | PERV-A | 8,918 | scaffold5028 | + | 23,883 | 32,747 |


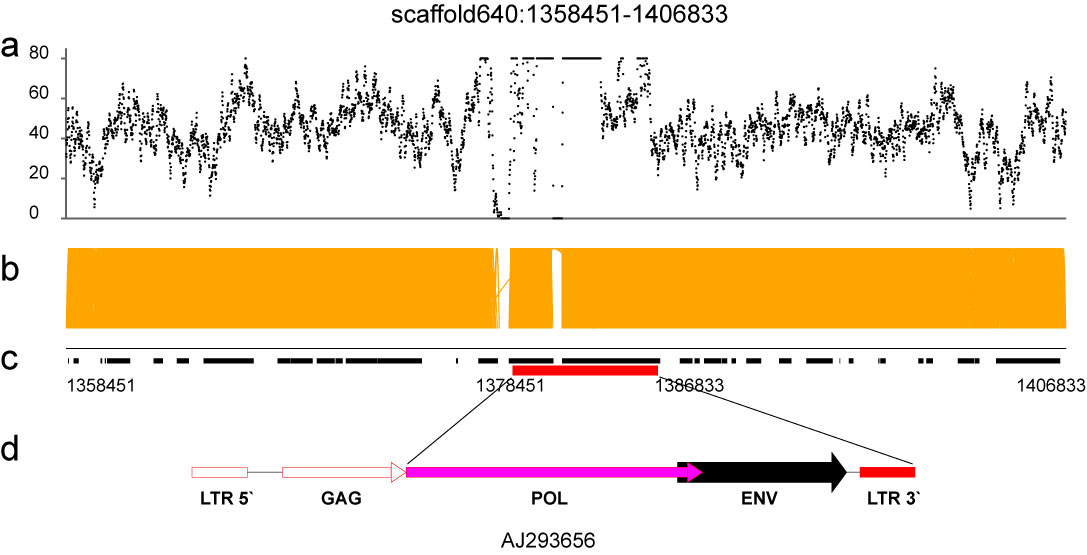


**Supplementary Fig. 5. Structure of PERV insertion in pig genome.** (a) Short insert read depth; (b) pair-end read coverage; (c) repeat content (black bar); (d) PERV gene structure.

# 5 Druggable domains and human disease related genes

Drug target gene information was downloaded from drugbank (http://www.drugbank.ca/). With orthologue information from human, macaque, mouse and pig, we can obtain the counterpart of each human drug target gene from the other species. Genes that lack orthologues in all three mammals were filtered out from our analysis. For orthologues that were lost in one or two species, the human drug gene protein was used to call on homologues in these gene synteny blocks, so as to confirm whether these genes were mis-assembled or mis-annotated. The mis-annotated genes were added.

Coronary artery disease (CAD) related genes of human were downloaded from CAD database (http://www.bioguo.org/CADgene/index.php). Using a similar procedure, we identified the orthologue of each angiocarpy related gene in the pig lineage. We then manually checked for genes that were lost or duplicated in the pig lineage.

**Supplementary Fig. 6. Drug target gene identity distribution between human and pig.**

**
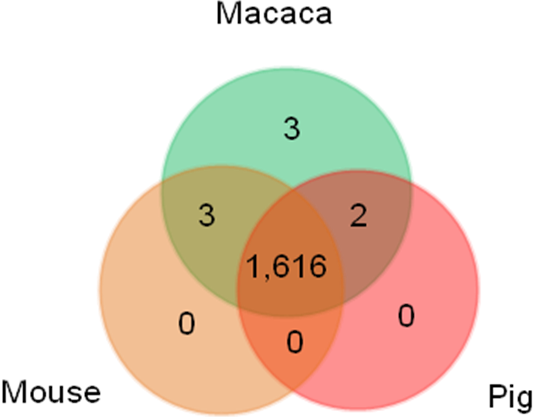
**

**Supplementary Fig. 7. Human drug target genes in macaque, mouse and pig.**

**Supplementary Fig. 8. *CYP3A4* post-translational modification sites in pig**

**Supplementary Fig. 9. Phylogenetic tree of tandem duplicated *CYP2J2* in relative species.**

**Supplementary Fig. 10. *FGA* and *FGB* residue changes in pig**

**Supplementary Fig. 11. *PAI-2*** **disulfide bond site lost in pig**

# 6 Supplementary references

1 Li, R. *et al.* De novo assembly of human genomes with massively parallel short read sequencing. *Genome Res* **20**, 265-272, (2010).

2 Li, H. & Durbin, R. Fast and accurate short read alignment with Burrows-Wheeler transform. *Bioinformatics* **25**, 1754-1760, (2009).

3 Li, H. *et al.* The Sequence Alignment/Map format and SAMtools. *Bioinformatics* **25**, 2078-2079, (2009).

4 Benson, G. Tandem repeats finder: a program to analyze DNA sequences. *Nucleic Acids Res* **27**, 573-580, (1999).

5 Chen, N. Using RepeatMasker to identify repetitive elements in genomic sequences. *Curr Protoc Bioinformatics* **Chapter 4**, Unit 4 10, (2004).

6 Kent, W. J. BLAT--the BLAST-like alignment tool. *Genome Res* **12**, 656-664, (2002).

7 Birney, E., Clamp, M. & Durbin, R. GeneWise and Genomewise. *Genome Research* **14**, 988-995, (2004).

8 Haas, B. J. *et al.* Improving the Arabidopsis genome annotation using maximal transcript alignment assemblies. *Nucleic Acids Research* **31**, 5654-5666, (2003).

9 Bairoch, A. & Apweiler, R. The SWISS-PROT protein sequence database and its supplement TrEMBL in 2000. *Nucleic Acids Res* **28**, 45-48, (2000).

10 Mulder, N. & Apweiler, R. InterPro and InterProScan: tools for protein sequence classification and comparison. *Methods Mol Biol* **396**, 59-70, (2007).

11 Ashburner, M. *et al.* Gene ontology: tool for the unification of biology. The Gene Ontology Consortium. *Nat Genet* **25**, 25-29, (2000).

12 Kanehisa, M. & Goto, S. KEGG: kyoto encyclopedia of genes and genomes. *Nucleic Acids Res* **28**, 27-30, (2000).

13 Li, H. *et al.* TreeFam: a curated database of phylogenetic trees of animal gene families. *Nucleic Acids Res* **34**, D572-580, (2006).

14 Kim, E. B. *et al.* Genome sequencing reveals insights into physiology and longevity of the naked mole rat. *Nature* **479**, 223-227, (2011).

15 Li, R. *et al.* The sequence and de novo assembly of the giant panda genome. *Nature* **463**, 311-317, (2010).

16 Yang, Z. PAML 4: phylogenetic analysis by maximum likelihood. *Mol Biol Evol* **24**, 1586-1591, (2007).

17 Yang, Z. & Nielsen, R. Codon-substitution models for detecting molecular adaptation at individual sites along specific lineages. *Mol Biol Evol* **19**, 908-917, (2002).

18 Zhang, J., Nielsen, R. & Yang, Z. Evaluation of an improved branch-site likelihood method for detecting positive selection at the molecular level. *Mol Biol Evol* **22**, 2472-2479, (2005).

19 Yang, Z. PAML: a program package for phylogenetic analysis by maximum likelihood. *Comput Appl Biosci* **13**, 555-556, (1997).
